# Detection of Microplastic in Salts Using Terahertz Time-Domain Spectroscopy

**DOI:** 10.3390/s21093161

**Published:** 2021-05-02

**Authors:** Jaeseung Im, Taewon Goo, Jugyoung Kim, Soobong Choi, Sung Ju Hong, Young-Mi Bahk

**Affiliations:** 1 Department of Physics, Incheon National University, Incheon 22012, Korea; sds1934@inu.ac.kr (J.I.); ergosum@inu.ac.kr (T.G.); 201600310a@gmail.com (J.K.); sbchoi@inu.ac.kr (S.C.); 2 Division of Science Education, Kangwon National University, Chuncheon 24341, Korea

**Keywords:** microplastic, terahertz time-domain spectroscopy, terahertz waves, effective medium model

## Abstract

We report on a prototypical study of the detection of microplastic embedded in table salts by using terahertz time-domain spectroscopy. In the experiment, high-density polyethylene (HDPE) of sizes from 150 to 400 μm are used as a representative microplastic and mixed with table salts. Analyzing terahertz transmittance with an effective medium model, we extract various optical properties such as refractive index, absorption coefficient, and real/imaginary parts of the dielectric constant of the mixture. Consequently, the optical properties exhibit volume-ratio-dependence in 0.1–0.5 THz regimes. Especially, the refractive index and the real part of the dielectric constant possess monotonic frequency dependence, meaning that the quantities can be relevant indicators for the detection of the microplastic in terms of practical applications. Our work proves that terahertz time-domain spectroscopy can pave a way to recognize microplastic mixed with salts and be expanded for detecting various micro-sized particles.

## 1. Introduction

Given severe threats not only to human beings but also the entire ecological system, microplastics have been recently paid attention [1,2,3]. Pollution by microplastics is ubiquitous within air [4], drinking water [5], and other substances [6,7,8]; for instance, it has been reported that table salt, an essential element for human metabolism, is contaminated by microplastics [6,9,10]. It is ultimately necessary to remove the microplastics so that intensive efforts have been carried out; for the first step, detecting the microplastic is a prerequisite [6,7,11,12,13,14]. Since plastics consist of non-conducting polymers, dielectric properties could be appropriate to distinguish them from surrounding environments [14]. In this regard, various optical properties in terms of spectroscopic methods, type/size of target material, and embedded medium have been investigated [6,13,15].

Due to the size of microplastics, generally, the wavelengths of incident light cover from μm (infrared light) to mm (terahertz waves), where the distinct wavelength is selectively employed for the purpose [16,17]. Representatively, Fourier transformed infrared spectroscopy and Raman spectroscopy have been used to identify and characterize microplastic itself because the wavelength is comparable with the size of microplastic [18,19]. In other words, while a similar length scale is relevant for the characterization of the particle, detecting in macroscale is time-consuming work. Compared to the aforementioned methods, terahertz spectroscopy is more relevant to probe within macroscopic scale [20]; namely, fast detection or imaging of the particle embedded in a large-sized medium is feasible. As a result, terahertz waves have been utilized for imaging, sensing, and estimating of desired materials [21,22]. Furthermore, time-domain experiment also provides both amplitude and phase information, enabling us to extract complex refractive index of the target materials [23,24,25]. As a result, the extracted refractive index and absorption coefficient have been employed to probe the desired materials [26]. In an aspect of practical application, measurement and analysis for the embedded structure of the microplastics in the surrounding medium are required, which are still lacking. A previous theoretical study can provide a basis to understand the optical properties of heterogeneous dielectric mixtures, where permittivity of the quasi-static effective medium is modeled [27]. Furthermore, most inhomogeneous mixtures are formed with powder type, which forces to construct a pellet-type structure. However, investigating the pellet-type sample is difficult to reflect realistic detection of various microplastics, requiring more practical exploration in terms of sample structure [19,28,29].

Here, we demonstrate an optical detection of microplastics in table salts by terahertz time-domain spectroscopy. A mixture of ground high-density polyethylene (HDPE) and table salts is used as a representative microplastic substance. To extract frequency-dependent optical parameters for each HDPE and salts, first of all, we analyze terahertz transmittance through each HDPE and salt particles embedded in the air using an effective medium model. Based on Landau-Lifshitz-Looyenga (LLL) model [30,31], we extract optical parameters of mixture samples in the terahertz frequency regime. As a result, the refractive index and the real part of the dielectric constant of mixture samples are strongly dependent on volume ratios between the HDPE and the entire mixture, enabling terahertz time-domain spectroscopy to probe microplastics in table salts. We expect that this work provides a prototypical base to investigate various complexes embedded in medium step by step and consequently macroscopic, fast, and non-destructive manner.

## 2. Materials and Methods

### 2.1. Preparation of HDPE, Table Salts, and the Mixture

**Figure 1:**
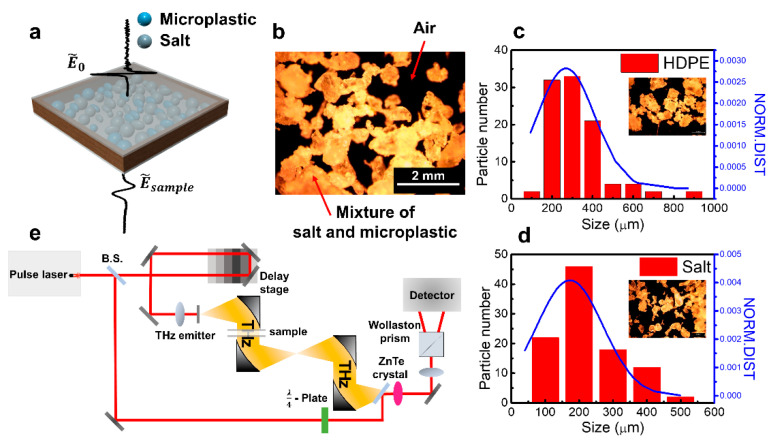
(**a**) An illustration of terahertz transmission measurement for the mixture of salt and microplastic (HDPE). (**b**) A representative optical image of the mixture (salt and HDPE) within the air. (**c**,**d**) Size distributions of HDPE (**c**) and salts (**d**). (**e**) Schematic of terahertz time-domain spectroscopy set up.

Figure 1a shows a schematic image of the HDPE/salt mixture in a holder and its terahertz transmission measurement. We note that the same holder with empty space is used as a reference for the terahertz transmittance. The corresponding optical image is presented in Figure 1b, where the mixture (HDPE:salt = 5:5) in the air was obtained by a dark-field optical microscope. Light and dark regions indicate the part of the mixture and air, respectively. The HDPE/salt mixture was prepared by filling the typical microplastics (HDPE, Koreapowder) and table salt (NaCl 99%, Hanju Corporation, Ulsan, Korea) in a home-made holder (50 × 50 × 3 mm^3^), where average sizes were 270 ± 140 μm and 170 ± 100 μm, respectively. The size distributions of HDPE/salts were measured in units of 100 μm by collecting the particles seen in a few optical images as shown in Figure 1c,d.

HDPE, table salts, and powder mixtures are packed in sample holders for terahertz transmission measurement. The front and rear sides of the holder are covered by a 1-mm-thick expanded polystyrene plate whose refractive index is assumed to be 1. To analyze terahertz data, we calculated volume fractions of powders and air by measured mass and known density of materials inside the holder, assuming the mass of air is zero. The volume of the inside holder is always $V_{inside}$ but volumes of HDPE and salt are $m_{H}/\rho_{H}$ and $m_{S}/\rho_{S}$, where m is mass and ρ is the density of each HDPE (H) and salt (S). The volume of air ($V_{air}$) can be calculated by $V_{inside}-m_{H}/\rho_{H}-m_{S}/\rho_{S}$. The volume fractions of the solid powder mixture and air are obtained by $V_{mixture}/V_{inside}$ and $V_{air}/V_{inside}$, where $V_{mixture}$ is the volume of the solid powder mixture.

### 2.2. Measurement and Analysis for Terahertz Time-Domain Spectroscopy

Terahertz time-domain spectroscopy was performed by using oscillator-based femtosecond Ti:sapphire laser with 780 nm center wavelength, 80 MHz repetition rate, and 100 fs pulse width (see schematic for the set up in Figure 1e). The laser was divided for generating and detecting terahertz pulses. Firstly, the one shine biased-photoconductive antenna (LT-GaAs) and consequently generates a single-cycle terahertz pulse. The terahertz pulse is guided by off-axis parabolic mirrors and illuminated on the sample. The terahertz pulse transmits through the sample and is finally detected in time-domain via electro-optic sampling method using 〈110〉-oriented 1-mm-thick ZnTe crystal with the rest femtosecond probe beam. We acquired amplitude and phase of the transmitted terahertz waves after fast Fourier transformation of time-domain results which are essential for calculating frequency-dependent refraction and extinction of the sample.

For observing the averaging effect of the large area on the sample, we positioned the sample on the collimated terahertz beam with a size of a few centimeters (Figure 1e). Four waveforms were averaged for each measurement. The empty space holder was measured as a reference before measuring powder samples. Time-domain signals are processed by fast Fourier transform for analyzing on frequency domain as mentioned. We determined the analyzing frequency range up to 0.5 THz possessing the significant amplitude signal of the sample: the amplitude is nearly zero due to the high extinction above 0.5 THz. Firstly, we obtained a complex refractive index of the mixture with air by using amplitude and phase of terahertz transmission. Subsequently, we excluded the air contribution by employing effective medium theory, where volume fractions between the mixture and air were considered with the LLL model. The detailed procedure for extracting the complex refractive index is presented below. Frequency-domain complex transmission $\tilde{t}\left(\omega \right)=\left|\tilde{t}\left(\omega \right)\right|e^{i\Delta \phi \left(\omega \right)}$ of sample is defined by a ratio between a field amplitude transmitted through a sample ${\tilde{E}}_{sample}\left(\omega \right)$ and that transmitted through a reference holder ${\tilde{E}}_{ref}\left(\omega \right)$ obtained by using fast Fourier transform of time-domain signals $E_{sample}\left(t\right)$ and $E_{ref}\left(t\right)$, respectively. We note that the pulse signals are pre-processed such as cutting and zero-padding effect before fast Fourier transformation. The complex refractive index of the sample ${\tilde{n}}_{s}\left(\omega \right)=n_{s}\left(\omega \right)-i\kappa_{s}\left(\omega \right)$, where $n_{s}$ and $\kappa_{s}$ are real and imaginary parts of refractive index, respectively, are extracted from the amplitude ($\left|\tilde{t}\right|$) and phase (Δϕ) data by using Fresnel equation, as following,

$$n_{s}\left(\omega \right)=1-\Delta \phi \left(\omega \right)\frac{c}{\omega d},$$

$$\kappa_{s}\left(\omega \right)=\frac{c}{\omega d}\ln\left(\frac{1}{\left|\tilde{t}\left(\omega \right)\right|}\frac{4n_{s}}{{\left(1+n_{s}\right)}^{2}}\right)$$

where d, ω, and c indicate thickness inside sample holder, angular frequency, and speed of light, respectively. 

The attenuation (α) related with absorption and scattering could be represented by using the equation $\alpha =2\kappa \frac{\omega }{c}$. In the experiment, the prominence between the scattering and absorption can vary with particle size in the inhomogeneous mixture. Since the scattering is the dominant factor compared to the absorption in extinction κ above 0.5 THz in our study, we present frequency-dependence up to 0.5 THz [22,32,33,34]. With the relation between refractive index and permittivity ($\tilde{n}=\sqrt{\varepsilon }$), the effective complex permittivity ($\varepsilon_{eff}$) is given by $\varepsilon_{eff}={\tilde{n}}_{s}{}^{2}$. For simplicity of measurement, the sample was prepared in the form of powder. Detecting materials with powder by analyzing permittivity requires considering the contribution of air within the holder. In this regard, we employed effective medium theories (EMT) which provide effective dielectric properties of the inhomogeneous constituent materials [27,35,36]. Basically, the EMTs depend on the volume fraction of the components of the mixtures [27,35]. Among various models including Maxwell-Garnet and Bruggeman model, the LLL model can be applied for our mixture powder samples which rely on not the shape, but the size of the constituent particles [37,38,39,40,41,42]. In other words, while the Maxwell-Garnet and Bruggeman models are suitable for the spherical shape of particles, the LLL model is more appropriate for the arbitrary shape of particles. Furthermore, the LLL model is appropriate for the case that the permittivity of the constituent materials has small contrast [27]. Since HDPE and table salts are randomly shaped, we adopt the LLL model for analyzing terahertz transmittance [31]. The effective dielectric constant extracted from the experiment, $\varepsilon_{eff}$, can be represented by $\varepsilon_{eff}^{1/3}=v_{1}\varepsilon_{air}^{1/3}+v_{2}\varepsilon_{mixture}^{1/3}$, where $v_{1}$ and $v_{2}$ are volume fractions of air and mixture (entire solid powder), $\varepsilon_{air}$ and $\varepsilon_{mixture}$ are complex dielectric constants of air and mixture, respectively.

## 3. Results and Discussion

**Figure 2:**
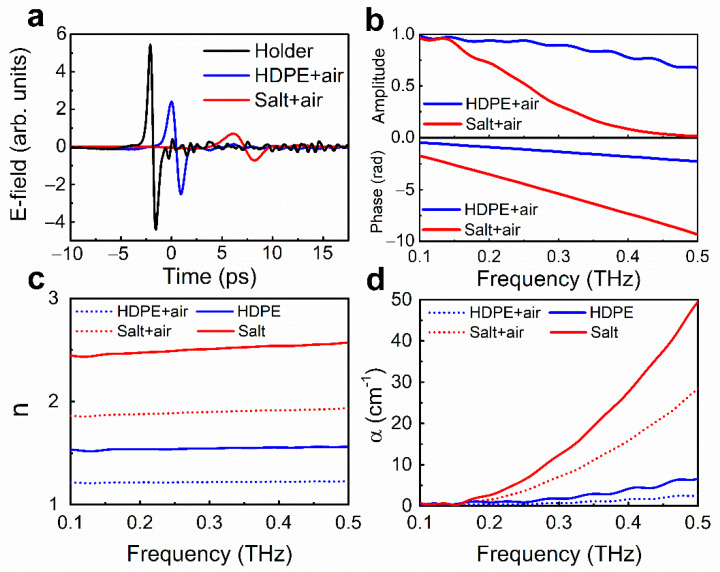
Extraction of refractive indices and absorption coefficients of HDPE and salt from terahertz transmittance. (**a**) Terahertz time traces of bare holder (black), HDPE-air (blue), and salt-air (red). (**b**) Amplitude (top) and phase (bottom) of HDPE-air (blue), and salt-air (red) normalized by the reference, as a function of frequency. (**c**,**d**) Extracted refractive indices (**c**) and absorption coefficients (**d**) of HDPE (blue) and salt (red), calculated by employing effective medium theory (LLL model). Dotted- and solid-lines correspond to cases of the samples before and after air consideration, respectively.

Figure 2a,b exhibit time traces and amplitude/phase spectra of terahertz transmission for pristine HDPE (blue) and table salt (red) with air, respectively. From the raw data, we extracted refractive index (n) and absorption coefficient (α) as shown in Figure 2c,d, respectively. Figure 2a shows time traces of the terahertz electric field transmitted through bare holder (black), HDPE (blue), and table salt (red). The results for HDPE and salt are delayed compared to that of air, indicating larger refractive indices (n>1). The field amplitudes decrease, and the waveforms stretch due to absorption and scattering dominant in high-frequency regimes originating from their material properties and size effects [19,28]. In particular, the long delay and more stretched pulse of the salt sample compared to that of HDPE implies a larger index and stronger extinction effect in high-frequency regimes. Figure 1b indicates amplitude (top) and phase (bottom) in the frequency domain. The absolute values of amplitude and phase in both cases decrease and increase with increasing frequency, respectively. From the data, we extracted frequency-dependent refractive index and absorption coefficient as shown in Figure 2c,d, respectively. The refractive index and absorption coefficient of HDPE-air and salt-air are depicted by blue- and red-dotted lines, respectively. In order to calculate optical properties of the pristine HDPE and salt, we adopted effective medium theory (LLL model), yielding the corresponding results with blue- and red-lines, respectively. It is commonly seen that the magnitudes of the refractive index and absorption coefficient of the pristine HDPE and salt are larger compared to each sample within the air, which can be attributed to averaging of the magnitudes in the air. The real part refractive indices of bulk HDPE and table salt extracted by the LLL model bring similar results with previous reports measured on pellet or bulk [30]. But the extinction parts are much higher than the results of pellet or bulk since we assume that loss caused by air effect is zero. Additionally, particle scattering is dominant in the attenuation factor.

**Figure 3:**
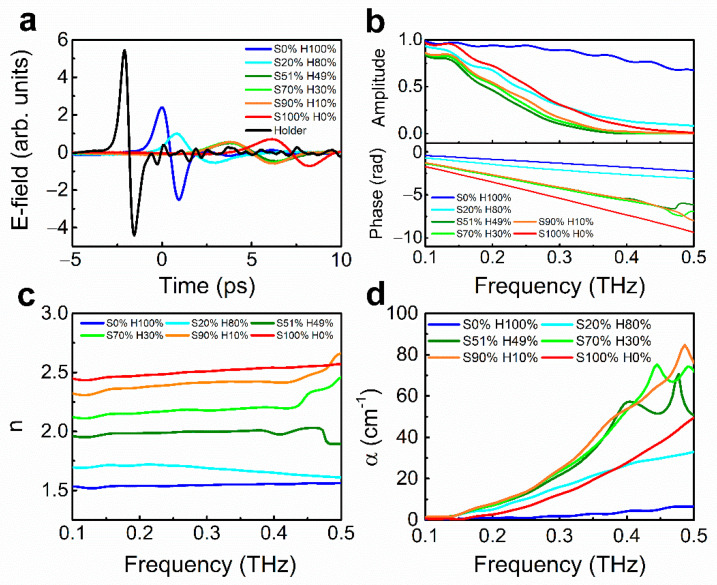
Extraction of refractive indices and absorption coefficients for a mixture of HDPE and salt with various compositions. (**a**) Terahertz time traces, (**b**) amplitude and phase spectra, (**c**) refractive indices, and (**d**) absorption coefficients for various mixtures of HDPE and salt, respectively.

Based on the pristine results, we investigate the mixture of HDPE/salt with various volume ratios in Figure 3. Similarly, we measured time traces of various compositions from 0 (100) to 100% (0%) of HDPE (salt) (Figure 3a) and plotted the corresponding field amplitudes and phases as shown in Figure 3b. In the graph, it is observed that amplitude spectra (upper part of Figure 3b) have a non-monotonic tendency with the volume ratio of HDPE, while phase differences (lower part of Figure 3b) show monotonic tendency relatively. The distinct features result in absorption coefficient (Figure 3d) and refractive index spectra (Figure 3c), respectively. It is noteworthy that all data of the mixture are corrected with effective medium theory for removing the contribution of air.

**Figure 4:**
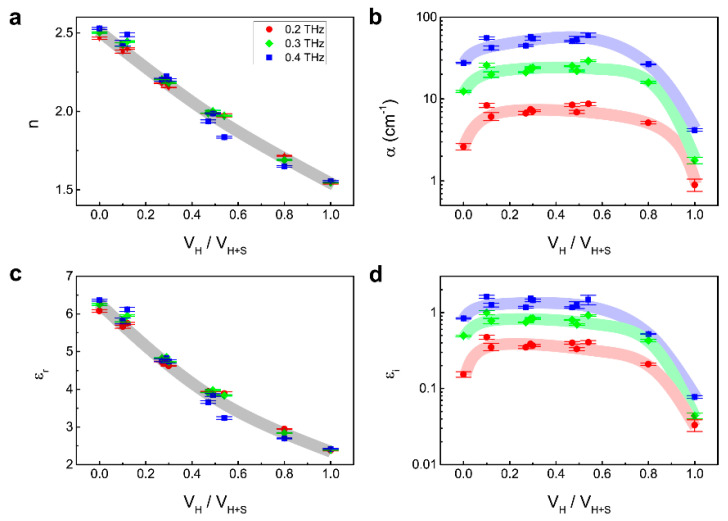
Optical indices as a function of volume ratio between HDPE and the entire mixture. (**a**–**d**) correspond to refractive index, absorption coefficient, the real and imaginary part of relative permittivity, respectively. Each line is a guide.

We replot the results as a function of the volume ratio between HDPE and the entire mixture at several frequencies (Figure 4). Figure 4a–d are (a) refractive index, (b) absorption coefficient, (c) real part, and (d) imaginary part of dielectric constant, respectively. For 0.2, 0.3, and 0.4 THz, monotonic changes are obtained in the case of refractive index and real part of dielectric constant, where negligible frequency-dependence is present. On the contrary, non-monotonic behaviors are observed in the case of absorption coefficient and imaginary part of dielectric constant also dependent on frequency. The frequency-dependent n and α of the mixture are understood by considering optical properties of the constituent materials: i.e., salt and HDPE exhibit the same behavior where n is frequency-independent and α has a larger value for higher frequency. For volume-ratio-dependence, α is likely to be sensitively affected by scattering due to geometry, shape, and distribution of constituent particles. The results imply that detection of microplastics (HDPE in this case) is more feasible with the refractive index or real part of dielectric constant due to its monotonic variation. We emphasize that this study provides a platform to detect microplastics in various materials. Even though we design the volume ratio of HDPE down to 10%, it does not mean the lowest detection limit; further experiments for a lower limit are necessary for future work. Furthermore, the substantial amount and size of microplastics in salts are much lower and smaller than this study, which can be overcome with a complementary study using the near-field imaging technique.

## 4. Conclusions

In summary, we demonstrated the monitoring of microplastics in table salts by terahertz time-domain spectroscopy. Using effective medium theory, we determined the refractive index and absorption coefficient of (i) each table salts and HDPE, and (ii) the mixture in powder type. For all cases, the refractive index is independent and the absorption coefficient increases with frequency, as usual. We observed that the refractive index monotonically changed with volume ratio, while the absorption coefficient sensitively varied with specific conditions due to the scattering effect in the powder type of the practical applicable sample. Based on the observation, we may suggest that detection of microplastics can be more feasible in terms of the refractive index rather than absorption coefficient in the terahertz frequency regime. This methodology, including a step-by-step analysis process, can be applied for various materials which form complex embedded in the medium.

## Data Availability

The data presented in this study are contained within the article.
